# Retrospective evaluation of prescribing pattern and utilization of antiepileptic drugs in pediatric, neurosurgery, and psychiatry wards: A comparative study to the standard treatment guidelines

**DOI:** 10.1097/MD.0000000000039818

**Published:** 2024-10-04

**Authors:** Marium Ayaz, Atif Ali, Rashida Bibi, Muhammad Mamoon Iqbal, Ayesha Iqbal, Sana Samreen, Wajid Syed, Hira Khan, Mahmood Basil A. Al-Rawi

**Affiliations:** a Department of Pharmacy, COMSATS University Islamabad, Abbottabad Campus, Abbottabad, Pakistan; b Department of Pharmacy, Abbottabad University of Science and Technology, Havelian, Abbottabad, Pakistan; c University Hospitals of Leicester, Leicester, United Kingdom; d Department of Pharmacy Practice and Policy, University Park Campus, University of Nottingham, Nottingham, United Kingdom; e Office of Lifelong Learning and the Physician Learning Program, Faculty of Medicine and Dentistry, University of Alberta, Edmonton, AB, Canada; f Department of Pharmacy, Aurobindo College of Pharmacy, Warangal, Telangana, India; g Department of Clinical Pharmacy, College of Pharmacy, King Saud University, Riyadh, Saudi Arabia; h Department of Optometry, College of Applied Medical Sciences, King Saud University, Riyadh, Saudi Arabia.

**Keywords:** antiepileptic drugs, first-generation antiepileptic drug, neurosurgery, pediatrics, psychiatry, sodium valproate

## Abstract

Antiepileptic drugs (AED) are progressively utilized for off-label conditions other than epilepsy, like bipolar disorder and migraine. The objective of this study was to evaluate current prescribing patterns and utilization of AED in pediatric, neurosurgery, and psychiatry wards and to compare them to the standard treatment guidelines. A descriptive, cross-sectional study was conducted in Ayyub Teaching Hospital, Abbottabad from December 1st, 2018 to April 2019. Data on demographic and clinical characteristics, utilization patterns of AED, adherence to standard treatment guidelines, and frequency of potential drug–drug interactions were analyzed using descriptive statistics. Among 410 patients, 54.3% (n = 223) were male, 45.6%(n = 187) were female, and 63.7% (n = 261) were from the 1 to 18 years’ age group. The majority 47.3% (n = 194) were from the pediatric ward followed by neurosurgery 28.7%(n = 118). Among the studied patients, 96.1% of them had comorbid conditions other than epilepsy alone. With regards to types of seizures unclassified seizures were the most common seizure type (59.8%; n = 245) followed by generalized tonic clonic seizures 23.4% (n = 96). In this study, the most frequently utilized AED was sodium valproate 59.0% (n = 242) followed by antiepileptic first-generation medicines were commonly used (76.3%). Although a total of 77.6% of the patients showed nonadherence to National Institute for Health and Care Excellence guidelines and 87.6% of them showed drug interactions. Findings from this study showed prescription patterns and utilization of AED in patients with epilepsy and non-epilepsy disorders which may help healthcare providers in making accurate clinical decisions.

## 1. Introduction

Epilepsy is a chronic noncommunicable life-threatening neurological disease of the brain associated with physical injuries^[1,2]^ and can be prevalent irrespective of age and gender.^[1,2]^ The World Health Organization reported that in 2023 approximately 50 million people worldwide have epilepsy Nearly 80% of people with epilepsy live in low- and middle-income countries.^[1]^ Furthermore, it is estimated that up to 70% of people living with epilepsy could live seizure-free if properly diagnosed and treated.^[1]^ In addition, premature death with epilepsy was 3 times higher than in the elderly and other populations.^[1]^ Despite this previous finding also revealed a high prevalence of 91.3% among school children.^[3]^ In Pakistan, a recent report revealed that the countrywide prevalence of epilepsy was 0.98%, and more than 2.2 million people have epilepsy in Pakistan.^[4]^ In 2022 the prevalence of generalized seizures among pediatric patients was reported at 90.7% (n = 205) while 9.3%(n = 21) of the children had focal seizures.^[5]^

Frequent seizures are characterized by epilepsy, which affects the brain.^[6]^ Even though seizures are brief bursts of electrical activity in the brain that momentarily alter how it functions.^[6]^ According to the International League against epilepsy 2017 There were different types of seizures among those focal onsets, followed by generalized onset seizures, which affect both sides of the brain at the same time and have unknown onset.^[7,8]^ Different types of seizures have different symptoms. However, the^[6–8]^ most common symptoms of epilepsy included staring, jerking movements of the arms and legs, loss of consciousness, breathing issues or stopping breathing, becoming stiff, falling suddenly for no apparent reason, especially when this was accompanied by loss of consciousness, temporarily not responding to noise or words, and strange sensations like a rising feeling in the stomach, strange smells or tastes, and a tingling sensation in the arms or legs.^[6,9]^

Furthermore, studies have shown that the majority of individuals with epilepsy have at least 1 comorbid disease. Neuropsychiatric illnesses, cognitive deficits, migraines, cardiovascular dysfunction, systemic autoimmune disorders, and other ailments are possible.^[10]^ According to the Epilepsy Foundation individuals with epilepsy are also more likely to have mood problems such as dysthymic disorder, anxiety and depression disorder, and bipolar disorder.^[11]^ Children with epilepsy are more likely to suffer from Attention Deficit Hyperactivity Disorder, which appears to be a prevalent problem in children with epilepsy. Attention Deficit Hyperactivity Disorder is present in one-fourth to one-third of children with epilepsy.^[11]^ Therefore, the treatment and prescribing pattern of antiepileptic drugs (AED) are very important in this situation. The treatment includes surgery to remove a small part of the brain that’s causing the seizures or a procedure to put a small electrical device inside the body that can help control seizures and the use of a ketogenic diet.^[6]^ While there were AED ranging from the oldest phenobarbital to the most recent Cenobamate and Cannabidiol is increasingly used to treat epilepsy.^[12]^ Furthermore, according to earlier research, the highly prescribed drugs were valproic acid and carbamazepine.^[13]^ The prescribing pattern of levetiracetam, phenytoin, and clobazam among epileptic patients was associated with adverse drug reactions.^[13,14]^ There have been studies that assessed the drug interactions and prescribing patterns of AED in other countries. The current data suggested that there were limited studies in this regard in Pakistan among the pediatrics, neurosurgery, and psychiatry. The objective of this study was to evaluate current prescribing patterns and utilization of AED in pediatric, neurosurgery, and psychiatry wards and to compare them to the standard treatment guidelines.

## 2. Methodology

### 2.1. Study design and setting

A descriptive, cross-sectional study was carried out in the pediatrics, neurosurgery, and psychiatry wards of Ayyub Teaching Hospital (abbreviated as ATH). ATH is a 1275-bed tertiary care teaching hospital located in Abbottabad, Khyber Pakhtunkhwa, Pakistan. It is an institute for education at the Undergraduate and Post Graduate levels in various disciplines of Medicine and Surgery. It is a health facility that is well-equipped with advanced diagnostic and therapeutic facilities.

### 2.2. Sampling technique and time frame of study

A convenience sampling technique was used for the data collection. The study was carried out for 4 months between December 2018 to April 2019. Patients visiting the study setting during the study duration and meeting the eligibility criteria were included in the study.

### 2.3. Sample size calculation

Similar to previous studies^[15–21]^ the sample size for this study was calculated using an online sample size calculator namely the Raosoft sample size calculator. The sample size was calculated at 95% confidence intervals and 5% margin of error, with an unknown population (n = 20,000) which yielded a sample of 377. Furthermore, to avoid sample bias and to improve the precision of the study we collected the data from 410 patients.

### 2.4. Inclusion and exclusion criteria

All patients in pediatric, neurosurgery, and psychiatry wards of both genders who received antiepileptic medications as a treatment irrespective of diagnosis, age, or gender were included in the present study. While pregnant/breastfeeding women were excluded from the present study.

### 2.5. Data collection

Data was collected by reviewing patient medical charts. The following details were extracted from patients’ medical records: Demographic characteristics such as gender, age, and respective ward. Clinical characteristics such as the presence of epilepsy with and without comorbid conditions, different types of comorbid conditions, and types of seizures. Data about the utilization pattern of AED included seizure-specific utilization of AED, utilization of AED in monotherapy or combination therapy, extent of individual antiepileptic drug utilization, generation of AED used and combination of AED with benzodiazepines. National Institute for Health and Care Excellence (NICE) guidelines were followed for comparing adherence to standard treatment guidelines. These guidelines provide treatment protocols for each type of seizure and are considered standard in clinical practice.^[22]^

### 2.6. Demographic characteristics

Patient demographic information was obtained from enrollment data which provided reasons for the eligibility of patients to be provided with healthcare-related services. It included information such as age, gender, and respective department.

#### 2.6.1. Clinical characteristics

According to NICE guidelines, the witness, history of the family, electroencephalography report, and epilepsy type were diagnosed. Opinions of concerned ward specialists/neurologists/psychiatrists were taken for diagnosis and treatment assessments. Also, this information was used in the seizure classification that also incorporated medical history and imaging studies. Types of seizures included: generalized tonic-clonic seizures, tonic seizures, focal seizures, myoclonic seizures, status-epilepticus, a combination of generalized and focal seizures, and unclassified seizures. Data about the presence of epilepsy with and without comorbid conditions was also analyzed.

#### 2.6.2. Utilization pattern of antiepileptic drugs

We measured exposure to antiepileptic medications based on brand name, generic name, and date when the prescription was filled. We examined seizure-specific utilization of AED, utilization of AED in monotherapy or combination therapy, the extent of individual antiepileptic drug utilization, and the combination of AED with benzodiazepines. Generations of AED were also included. First- and second-generation antiepileptic medications included: barbiturates (phenobarbital), benzodiazepines (clonazepam, diazepam, lorazepam), hydantoin (phenytoin), succinimides (ethosuximide), acetazolamide, valproic acid, and carbamazepine. Third-generation antiepileptic medications included: levetiracetam, pregabalin, and topiramate.

### 2.7. Comparison to the standard treatment guidelines

For the assessment of the rationality of prescriptions, each case was evaluated during the study in comparison with the standard guidelines. AED prescribed were compared to the standard treatment guidelines (NICE guideline) to evaluate whether AED are prescribed as per NICE guidelines or not.

### 2.8. Analysis of potential DDIS

Potential drug-drug interactions (DDIS) among AED and medications concomitantly prescribed for other comorbid conditions were analyzed by using the Lexi-Interact program of Lexi comp® (Version 4.4.1), a drug information database. This database classifies interactions based on severity such as major, moderate, and minor drug interactions. The risk rating is labeled in Lexi comp as avoid the combination, modify treatment regimen, monitor therapy, no action needed, and no interactions with the coding of X, D, C, B, and A, respectively.^[23–25]^

### 2.9. Statistical analysis

Statistical analysis was performed by using Statistical Package for Social Sciences (IBM SPSS Statistics V24.0). To present data, descriptive statistics such as percentage (%) and frequencies (n) were used in which data was presented in frequencies and percentages.

### 2.10. Ethical consideration

This study was approved by the ATH, Abbottabad and Research Ethics Committee, Department of Pharmacy, COMSATS University Islamabad, Abbottabad campus. Verbal consent was taken from patients in the language that they understood.

## 3. Results

Data from 410 patients admitted in the pediatric, psychiatry, and neurosurgery wards who were prescribed different antiepileptic medications was gathered and evaluated.

### 3.1. Demographic characteristics

The current study had a male-to-female ratio of 2:1, with 223 (54.3%) male and 187 (45.6%) female patients. In this study, 261 (63.7%) cases were between the ages of 1 and 18, 102 (24.9%) were 19 and 45 years, and 47 (11.5%) were aged between 46 and 60 years old. In comparison to neurosurgery and psychiatry wards, the majority of the study patients came from 194 (47.3%) pediatric wards. Table 1 displays demographic information.

**Table 1:** Demographic characteristics, epilepsy and comorbid conditions and drug interactions.

Variables	Frequency (%)
**Gender**	
Male	223 (54.3**%**)
Female	187 (45.6**%**)
**Age distribution (years**)	
1–18	261 (63.7**%**)
19–45	102 (24.9**%**)
46–60	47 (11.5**%**)
**Ward name**	
Pediatric	194 (47.3**%**)
Neurosurgery	118 (28.7**%**)
Psychiatry	98 (23.9**%**)
**Diagnosis**	
Epilepsy with comorbidity	395 (96.1**%**)
Epilepsy without comorbidity	15 (3.9**%**)

### 3.2. Clinical characteristics

In terms of comorbidities, 395 (96.1%) patients had comorbid epilepsy, while 15 (3.9%) patients were only epileptic, as shown in Table 1. Unclassified seizures were the most common, accounting for 245 (59.8%), followed by 96 (23.4%) generalized tonic-clonic seizures, 33 (8.0%) focal seizures, 20 (4.9%) status epilepticus, 9 (2.2%) tonic seizures, 6 (1.5%) combination of focal seizures and generalized seizures, and 1 (0.22%) myoclonic seizures, as shown in Table 2.

**Table 2:** Clinical characteristics.

Variables	Frequency (n)	Percentages (%)
**Types of seizure** Generalized tonic-clonic seizures Focal seizures Tonic seizures Myoclonic seizures Focal seizures + generalized seizures Status epilepticus Unclassified seizures	96 33 9 1 6 20 245	23.4% 8.0% 2.2% 0.22% 1.5% 4.9% 59.8%
Treatment regimen (monotherapy/polytherapy)	169/241	41.2%/58.8%
**Generation of antiepileptic’s** First generation antiepileptics Second generation antiepileptics First generation + second generation First generation + second generation + third generation First generation + third generation	313 9 81 6 1	76.3% 2.2% 19.8% 1.5% 2%
NICE Guidelines (followed/not followed)	92/318	22.4%/77.6%
Drug interactions (present/not present)	359/51	87.6%/12.4%
Type of drug interaction (major/moderate/minor)	135/462/33	21.3%/73.1%/5.2%

It demonstrated that there were fewer epileptic AED users than non-epileptic AED users. Meningitis accounted for 193 (47.1%) cases of comorbid conditions, with encephalitis being the most frequently identified CNS infection. Next, 111 (27.1%) cases of neurosurgical conditions like space-occupying lesions and gliomas, 89 (21.7%) cases of psychiatric conditions like bipolar affective disorder with mania, anxiety, and depression, and 2 (0.4%) cases of dysentery and acute gastroenteritis followed (Table 3).

**Table 3:** Different types of comorbid conditions.

Comorbid conditions	Frequency (n)
Meningitis, encephalitis	193 (47.0%)
Dysentery, acute gastroenteritis	2 (0.4%)
Bipolar affective disorder with mania	89 (21.7%)
Space-occupying lesions, glioma	111 (27.0%)

### 3.3. Utilization pattern of antiepileptic medications

Table 4 demonstrated that (51.5%) diazepam was used to treat GTC seizures, (33.3%) lacosamide for treating focal seizures, (11.1%) lorazepam for treating tonic seizures, (25.0%) Topiramate for treating combined focal and generalized seizures, (11.1%) lorazepam for treating myoclonic seizures, (33.3%) lacosamide for status epilepticus, and 82.9% clonazepam for treating unclassified seizures.

**Table 4:** Seizure-specific utilization pattern of antiepileptic drugs.

Utilization pattern of AED	GTC seizures (%)	Focal seizures (%)	Tonic seizures (%)	Focal seizure + generalized seizure (%)	Myoclonic seizure (%)	Status epilepticus (%)	Unclassified seizures (%)
Phenytoin	42.5	13.2	3.8	5.7	0.0	13.2	21.7
Carbamazepine	15.2	6.1	3.0	3.0	0.0	0.0	72.7
Diazepam	51.5	11.3	5.2	3.1	0.0	10.3	18.6
Levetiracetam	36.3	14.7	4.9	3.9	0.0	10.8	29.4
Phenobarbitone	38.9	14.8	7.4	1.9	0.0	5.6	31.5
Clonazepam	9.8	0.0	1.2	1.2	0.0	4.9	82.9
Midazolam	33.8	14.3	0.0	3.9	0.0	10.4	37.7
Lorazepam	0.0	0.0	11.1	0.0	11.1	0.0	77.8
Sodium Valproate	38.3	9.6	4.2	3.0	0.0	6.6	38.3
Topiramate	0.0	0.0	0.0	25.0	0.0	0.0	59.6
Lacosamide	16.7	33.3	0.0	16.7	0.0	33.3	0.0
Vigabatrin	1.0	0.0	0.0	0.0	0.0	0.0	0.0

AED = antiepileptic drugs.

A total of 241 (58.8%) patients were treated by using 2 or more 2 AED while 169 (41.2%) patients were treated by using a single antiepileptic drug as described in Table 5.

**Table 5:** Utilization of AED in monotherapy or combination therapy.

Treatment regimen	No AED per prescription	n (%)
Monotherapy	1 AED	169 (41.2%)
Combination therapy	2 or >2 AED	241 (58.7%)

AED = antiepileptic drugs.

**Figure 1.**
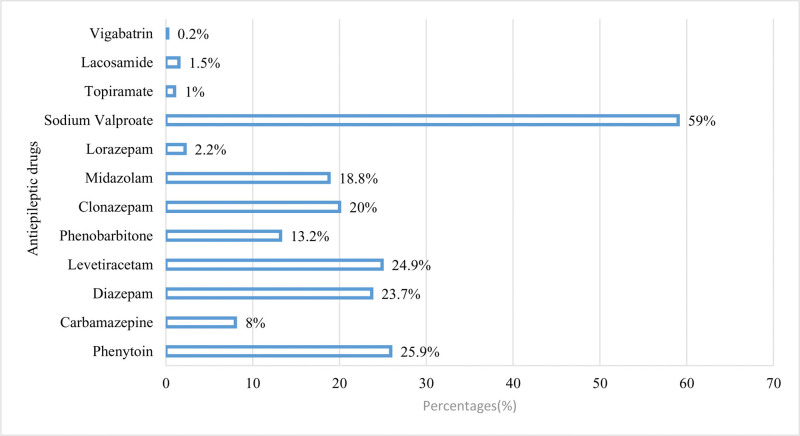
Extent of antiepileptic drug utilization.

Figure 1 showed that for epilepsy management 242 (59.0%) sodium valproate was frequently utilized followed by 106 (25.9%) phenytoin, 102 (24.9%) levetiracetam, 97 (23.7%) diazepam, 82 (20.0%) clonazepam, 77 (18.8%) midazolam, 54 (13.2%) Phenobarbitone, 33 (8.0%) carbamazepine, 6 (1.5%) lacosamide, 4 (1.0%) Topiramate, 1 (0.2%) vigabatrin.

**Figure 2.**
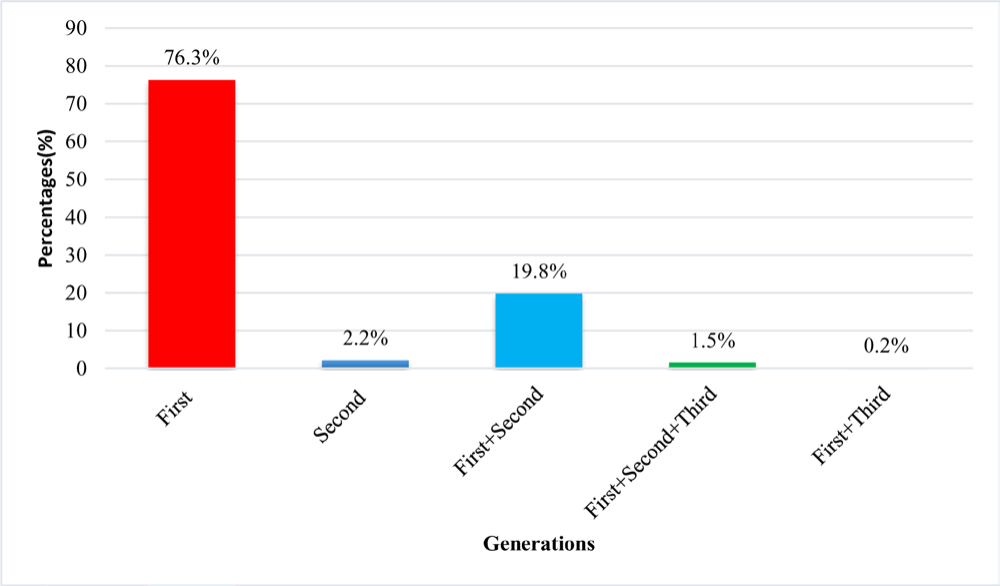
Generation of antiepileptic drugs used.

In this study, 76.3% (n = 313) of the first-generation antiepileptic’s were frequently used followed by 81 (19.8%) combinations of first and second-generation antiepileptic’s, 9 (2.2%) second-generation antiepileptic, 6 (1.5%) combination of first, second and third generation 6 (1.5%) and 1 (0.24%) combination of first and third generation antiepileptic’s as shown in Figure 2.

**Figure 3.**
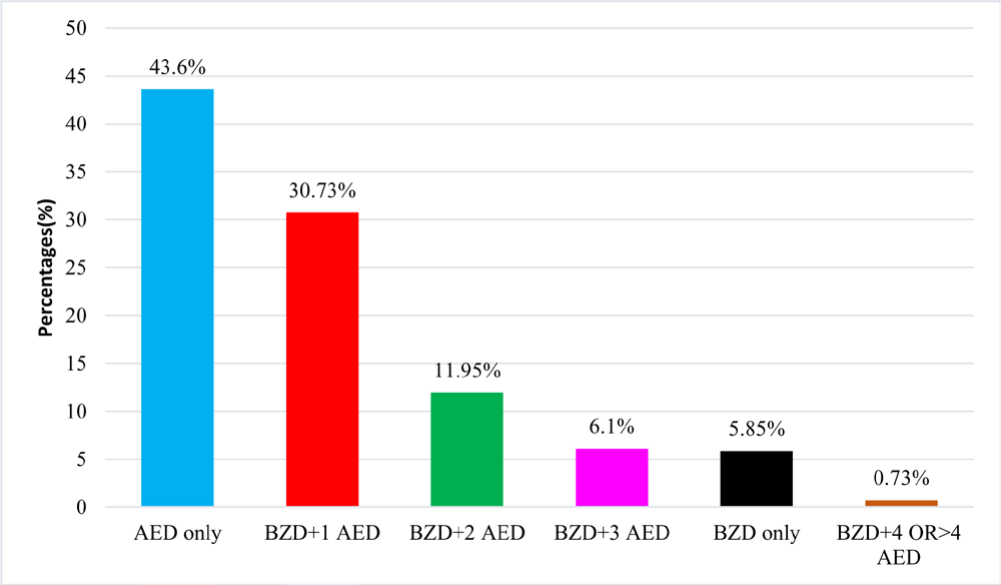
Utilization of AED and benzodiazepine combination. AED = antiepileptic drugs.

In this study 179 (43.7%) cases were treated by using an AED only, 126 (30.7%) cases were treated by using benzodiazepine with 1 AED, 49 (12.0%) cases were treated by using benzodiazepines with 2 AED, 25 (6.1%) cases were treated by using benzodiazepine with 3 AED, 24 (5.9%) cases were treated by using benzodiazepine drug only, 3 (0.73%) cases were treated by using benzodiazepines with 4 or more than 4 AED as described in Figure 3.

### 3.4. Adherence to standard guidelines

Out of 410 cases, it was found that 77.6% did not adhere to standard treatment guidelines (NICE). In 22.4% of cases, treatment guidelines were followed as given in Table 6

**Table 6:** Adherence to standard guidelines, prevalence, and severity of potential DDIS.

Variables	Frequency (n)	Percentage (%)
**NICE guidelines**		
Not followed	318	77.6%
Followed	92	22.4%
**Drug interactions**		
Present	359	87.6%
Not present	51	12.4%
**Severity**		
Major	135	21.3%
Moderate	462	73.1%
Minor	33	5.2%

### 3.5. Prevalence of potential for DDIS

Out of 410 patients, potential drug interactions were identified in 359 (87.6%) patients as described in Table 6.

### 3.6. Severity of drug interactions

A total of 632 DDIS were identified in the present study. Table 3 showed that the majority of interactions were of 462 (73.1%) moderate type followed by 135 (21.3%) major type and 33 (5.2%) minor type (Table 7).

**Table 7:** Frequency of moderate, major, and minor DDIS.

Moderate drug interactions	Frequency (%)
Haloperidol–procyclidine	73 (17.8%)
Paracetamol–phenytoin	59 (14.3%)
Clonazepam–haloperidol	53 (12.9%)
Levetiracetam–phenytoin	47 (11.4%)
Diclofenac–dexamethasone	43 (10.4%)
Levetiracetam–midazolam	37 (9.0%)
Diazepam–levetiracetam	34 (8.2%)
Levetiracetam–phenobarbitone	33 (8.0%)
Furosemide–phenytoin	32 (7.8%)
Ranitidine–phenytoin	31 (7.5%)

Major drug interactions	Frequency(%)
Ceftriaxone–calcium gluconate	59 (14.3%)
Diazepam–phenytoin	52 (12.6%)
Midazolam–phenytoin	32 (7.8%)
Phenytoin–dexamethasone	31 (7.5%)
Midazolam–phenobarbitone	25 (6.0%)
Clonazepam–carbamazepine	22 (5.3%)
Clonazepam–olanzapine	21 (5.1%)
Diazepam–metronidazole	13 (3.1%)
Dexamethasone–phenobarbitone	13 (3.1%)
Diazepam–phenobarbitone	12 (2.9%)
Sodium valproate–meropenem	11 (2.6%)

Minor drug interactions	Frequency (%)
Sodium valproate/clonazepam	52 (12.6%)
Diazepam/sodium valproate	27 (6.5%)
Paracetamol/metoclopramide	26 (6.3%)
Paracetamol/tramadol	9 (2.1%)
Lacosamide/phenobarbitone	3 (0.7%)
Phenytoin/sodium bicarbonate	3 (0.7%)
Ondansetron/albuterol	2 (0.4%)
Chlorpheniramine maleate/ondansetron	2 (0.4%)
Ranitidine/sodium bicarbonate	2 (0.4%)
Nimodipine/sodium valproate	2 (0.4%)

### 3.7. Moderate DDIS

Table 7 showed that in moderate interaction 73 (17.8%) haloperidol/procyclidine was the most frequent moderate drug interaction followed by 59 (14.3%) paracetamol/phenytoin and 53 (12.9%) clonazepam/haloperidol.

### 3.8. Major DDIS

In Table 7 major drug interactions have been enlisted. Fifty-nine (14.3%) cases had ceftriaxone-calcium gluconate interaction followed by 53 (12.6%) diazepam-phenytoin and 32 (7.8%) midazolam-phenytoin.

### 3.9. Minor DDIS

Table 7 showed a list of minor interactions among which 52 (12.6%) sodium valproate/clonazepam was the most frequently occurring minor interaction followed by 27 (6.5%) diazepam/sodium valproate and 26 (6.3%) paracetamol/metoclopramide.

## 4. Discussion

The irrational prescription of medicines exists everywhere throughout the world furthermore, in the long run, they lead to undesirable impacts in patients.^[26–28]^ In this examination, the current prescribing patterns and utilization of antiepileptic medications in a tertiary care teaching hospital in Abbottabad were evaluated. The discoveries from this examination are essential to the healthcare settings of Pakistan since they help to evaluate whether the ATH is following a set standard of practices to guarantee optimal medication utilization. Few studies are accessible from Pakistan on this issue and along these lines, our discoveries provide an insight into the persistent monitoring of medication treatment and procedure advancement at the institutional level. Notwithstanding the contribution made in the Pakistani setting, our discoveries may likewise be pertinent to other nations with comparable medication use practices or with comparative healthcare settings. This investigation may give the motivation for scholars, clinicians, and those managing hospital administration in those nations to start to survey the status of their own countries prescribing antiepileptic’s inside pediatric, neurosurgery, and psychiatry wards.

The present study showed a higher male-to-female ratio of 2:1. Two hundred twenty-three (55.4%) were male patients and 187 (45.6%) were female patients. It showed a higher percentage of male patients as compared to female patients. This result is in agreement with the previous study which shows that the study population had more male patients as compared to female patients.^[29]^ The proportion of males is greater than females in the Pakistani patient population which is by many other examinations.^[30]^ A study reports a greater proportion (55.9%) of male patients as compared to (44.1%) of female patients because those males are more vulnerable to the risk factors of epilepsy of lesion type and seizures of acute symptomatic type.^[31,32]^ In the current study, 261 (63.7%) cases are in the 1–18 age group, 102 (24.9%) in the 19–45 age group, and 47 (11.5%) in the 46–60 + age group. The age categories in the current study population are rather comparable to those previously identified in Colombia, Italy, and the United Kingdom.^[33]^ However, the present study is not compatible with earlier studies, which found that epilepsy is more prevalent between 20 to 40 years of age.^[34]^

Comorbid ailments, such as depression and psychosis, are more prevalent in epileptic patients and indicate a higher risk of neurological disorders.^[35]^ In this study, 395 (96.4%) of the patients had comorbid epilepsy, while 15 (3.7%) exclusively had epilepsy. It demonstrated that the number of epileptic AED users was lower than that of non-epileptic AED users. The comorbid conditions that occurred most commonly were meningitis, encephalitis, and tuberculous meningitis accounting for 193 (47.1%) cases, which are the most common CNS infections. Neurosurgical conditions such as space-occupying lesions and gliomas account for 111 (27.1%) cases, while psychiatric conditions such as bipolar affective disorder with mania, anxiety, and depression account for 89 (21.7%) cases. In addition, meningitis and tuberculous meningitis are the 2 most frequent CNS ailments, accounting for 62.6 % of all epilepsy cases. According to a previous study, Pakistan has more than 400,000 tuberculosis cases each year, making it the world’s fifth largest. It has also been estimated that around 10% of tuberculosis patients have involvement of the central nervous system, and the incidence of CNS-related tuberculosis is proportionate to the prevalence of tuberculosis-related infections^[36]^ and Comorbidities occur at a (46%) higher incidence.^[37]^This inquiry is further supported by the findings of many other epidemiological exams that also documented the high prevalence of most prevalent comorbid conditions in epileptic patients as compared to unaffected ones.^[38]^

Seizures are broadly perceived as the clinical sign of epilepsy.^[39]^ It is necessary to make a correct diagnosis of seizures because the efficacy of AED is affected by the seizure type for which it is used, comorbid conditions, and adverse effects.^[40]^ In the current study, unclassified seizures were most common 245 (59.8%) followed by generalized tonic-clonic seizures 96 (23.4%), focal seizures 33 (8.0%), status epilepticus 20 (4.9%), tonic seizures 9 (2.2%), a combination of focal seizures and generalized seizures 6 (1.5%) and myoclonic seizures 1 (0.22%). According to a previous study, 245 (72.91%) generalized tonic-clonic seizure is the most common seizure type as compared to 85 (25.9%) unclassified seizures.^[41]^ The important limitation in the present study is the presence of case notes/prescriptions of patients with epilepsy in which the seizure type was unclassified or not recorded. This problem was either due to the inability of healthcare providers to make correct diagnoses or to properly record and maintain the patient’s medical records. It was found in another study that 61.7% of partial seizures are more common followed by 40.7% generalized seizures, and 0.2% unclassified seizures.^[42]^

The present study showed that (51.5%) diazepam was used to treat GTC seizures, (33.3%) lacosamide for focal seizures, (11.1%) lorazepam for tonic seizures, (25.0%) Topiramate for combined focal and generalized seizures, (11.1%) lorazepam for myoclonic seizures, (33.3%) lacosamide for status epilepticus and 82.9% clonazepam for unclassified seizures treatment. A previous study reports that (21.3%) sodium valproate frequently is used for the treatment of generalized tonic-clonic seizures because of its broad spectrum antiepileptic activity it is used as a first-line drug or as an add-on drug for the treatment of seizures of generalized type.^[40]^

Research related to drug utilization adds to the rational utilization of medications by reporting medication use patterns and intercession.^[43]^ In the present study, the antiepileptic drug utilization pattern was assessed to check whether the AED were used in monotherapy (single AED only) or combination therapy (2 or more than 2 AED) for the management of epilepsy and non-epilepsy disorders. The results of the study showed that the majority of the patients were treated with antiepileptic drug polytherapy. Two hundred forty-one (58.8%) patients were treated by using 2 or more 2 AED while 169 (41.2%) patients were treated by using a single antiepileptic drug. Monotherapy is mostly preferred over polytherapy if possible, but all patients on 1 AED will not achieve complete seizure control. Polytherapy may lead to an increase in the risk for adverse reactions as well as potential DDIS between antiepileptic or other concomitantly administered drugs.^[44]^ For the treatment of seizures, monotherapy was used in 80% of cases.^[45]^ Monotherapy is suitable because a single AED can control seizures and is associated with fewer side effects. As compared to polytherapy, monotherapy is preferred due to less cost associated with medicine use, reduction in adverse effects, potential DDIS, and enhanced medicine compliance.^[46]^

Findings of the present study showed that for epilepsy management sodium valproate was frequently utilized 242 (59.0%) followed by phenytoin 106 (25.9%), levetiracetam 102 (24.9%), diazepam 97 (23.7%), clonazepam 82 (20.0%), midazolam 77 (18.8%), phenobarbitone 54 (13.2%), carbamazepine 33 (8.0%), lacosamide 6 (1.5%), topiramate 4 (1.0%), vigabatrin 1 (0.2%), 198 (53.1%) sodium valproate is the most commonly used antiepileptic drug used for epilepsy management. Seventy-nine (39.4%) sodium valproate is frequently used for the epilepsy treatment.^[47]^ A previous study reports that AED that is commonly used is (16.8%) sodium valproate because of its broad spectrum antiepileptic effect and is the drug of choice for the treatment of generalized seizures and to some extent for focal seizures.^[40]^ First-line AED for the treatment of generalized tonic-clonic seizures are sodium valproate, lamotrigine, and topiramate. Due to this reason, sodium valproate is the most commonly utilized AED for the management of seizures of generalized tonic-clonic type.^[48]^

AED are classified as first-generation (valproic acid, carbamazepine), second-generation (levetiracetam, topiramate), and third-generation (lacosamide, eslicarbazepine) AED.^[44]^ In the current study majority of antiepileptic medications were first-generation antiepileptics 313 (76.3%), followed by a combination of first and second-generation 81 (19.8%), second-generation antiepileptics 9 (2.2%), a combination of first, second and third generation 6 (1.5%), combination of first and third generation 1 (2%). A previously reported study shows that in 2527 (54%) patients first-generation AED are used to initiate epilepsy treatment and management. Newer generation (Second and Third generation AED) AED cannot be used for the treatment because very little data is available on their clinical effectiveness, cost issue and they are preferred to be used in those epileptic patients who do not respond to treatment with older/conventional (first generation AED) AED.^[46]^

AED are used in combination with benzodiazepines such as clonazepam for the treatment of seizures.^[49]^ Present study findings showed that 179 (43.7%) cases were treated by using antiepileptic only, 126 (30.7%) cases were treated by using benzodiazepine with 1 antiepileptic drug, 49 (12.0%) cases were treated by using benzodiazepines with 2 AED, 24 (5.9%) cases were treated by using benzodiazepines only as well benzodiazepines with 3 antiepileptic, 4 (1.0%) cases were treated by using benzodiazepines with 4 or more than 4 AED. A previously reported study shows that 61.9% of cases used AED only and 28.6% used a combination of AED with 1 antiepileptic.^[49]^ Another previous study reports 51.5% of AED use only and 40.62% use of antiepileptic with benzodiazepine.^[50]^ One more study reports the use of (22.4%) AED only and (20.5%) AED with 1 benzodiazepine.^[49]^ Due to the anticonvulsant properties of benzodiazepines, they can be used for the control of convulsions. They are effective as anticonvulsants but when administered for longer periods result in tolerance and side effects including sedation. They are considered adjuvant medication to the standard antiepileptic medications where they have failed to give control.

### 4.1. Adherence to NICE guidelines

NICE ought to not simply be seen as a reference guideline for best practice but as an urgent need for rebuilding services for individuals with epilepsy.^[51]^ The findings of the present study revealed that in 318 (77.6%) cases drugs were not prescribed as per NICE guidelines for epilepsy treatment while in only 92 (22.4%) cases drugs were prescribed as per NICE guidelines for the treatment of epilepsy. Nonadherence to the standard treatment guidelines shows that either those guidelines are unavailable or the lack of knowledge and inability of prescribers to make correct diagnoses about seizure classification and then prescribing medication according to that diagnosis. A previously reported study implies that in 80% of cases AED are prescribed according to the NICE guidelines for the management of seizures.^[45]^ Such recommendations offer an outline on which practice needs to be based and guarantee that equal principles of care are set throughout all regions.^[51]^

### 4.2. Potential DDIS

Few studies are available on the drug interactions between AED and other concomitantly administered drugs.^[52]^ Present study findings revealed that 462 (73.1) moderate drug interactions were the most common type of potential DDIS followed by 135 (21.3%) major drug interactions, 33 (5.2%) minor drug interactions, and those drug interactions in which severity was not available 2 (0.3%). A previous study finding shows that 32% of cases had major drug interactions. Patients admitted to the hospital showed an increase in the risk of DDIS due to the presence of several comorbid conditions and the drugs used to treat those comorbid conditions in the form of polytherapy.^[53]^ The present study findings revealed that AED have shown interactions with other drug classes like antibiotics, antiprotozoal, antituberculosis drugs, antifungal, anthelmintic, antiviral, proton-pump inhibitors, H2-receptor antagonists, antiarrhythmic, lipid-lowering drugs, antipsychotics, benzodiazepines, non-steroidal anti-inflammatory drugs, and corticosteroids. A previous study reports an interaction between AED and other drug classes similar to the present study results. In previous years, interactions of AED were identified most of the time by coincidence, but now at this level, improvements have been made due to advancements in the concept of enzyme induction and inhibition. Due to this, it is now possible to manage such types of interactions.^[52,53]^

Although this study has several limitations, the first is that the data was collected from a single teaching hospital, which limits its relevance to other settings or areas. Second, the study is short in time frame, which may not represent long-term trends in AED consumption. As a result, to establish a higher level of evidence, it is proposed that a study with larger representative samples from several hospitals throughout the country be conducted. Furthermore, programs to improve healthcare professionals’ knowledge of AED prescribing should be developed. Our findings highlight the importance of developing policies that enable equal access to the most appropriate AED for all patients, regardless of socioeconomic level.

## 5. Conclusion

In this study, 77.6% of the patients showed non to NICE guidelines and 87.6% of them showed drug interactions. Nonadherence to NICE guidelines may be due to the unavailability of guidelines or the inability of prescribers to make correct diagnoses. Major and moderate drug interactions were more prevalent than minor ones. AED were used to treat both epilepsy and non-epilepsy disorders. The adherence status and drug interactions were measured based on a patient chart which is prone to recall and social desirability bias. Unclassified seizures were more common than GTC and other seizure types. Sodium valproate was the most frequently utilized AED involving the use of first-generation AED. Study about the prescription patterns of AED over time contributes to the aspects related to drug safety/pharmacovigilance as it provides baseline information for those authorities who make decisions at the national level about the utilization of medications, for improving standard treatment guidelines and for monitoring those risks (adverse effects and drug interactions) related to the utilization of medications in a patient population. The findings of this study can be used by healthcare providers and those associated with policymaking to improve medication utilization in healthcare settings.

## Acknowledgments

The authors of this study extend their appreciation to the Researchers Supporting Project (Project number RSPD2024R1099), King Saud University, Riyadh, Saudi Arabia.

## Author contributions

**Conceptualization:** Rashida Bibi, Muhammad Mamoon Iqbal, Ayesha Iqbal, Wajid Syed, Hira Khan, Mahmood Basil A. Al-Rawi.

**Data curation:** Atif Ali, Rashida Bibi, Muhammad Mamoon Iqbal, Hira Khan, Mahmood Basil A. Al-Rawi.

**Formal analysis:** Marium Ayaz, Atif Ali, Rashida Bibi, Muhammad Mamoon Iqbal, Sana Samreen, Wajid Syed, Hira Khan, Mahmood Basil A. Al-Rawi.

**Funding acquisition:** Wajid Syed.

**Investigation:** Atif Ali, Rashida Bibi, Muhammad Mamoon Iqbal, Hira Khan.

**Methodology:** Marium Ayaz, Rashida Bibi, Muhammad Mamoon Iqbal, Ayesha Iqbal, Sana Samreen, Wajid Syed, Hira Khan.

**Project administration:** Marium Ayaz, Rashida Bibi, Ayesha Iqbal, Wajid Syed, Hira Khan.

**Resources:** Rashida Bibi, Muhammad Mamoon Iqbal, Ayesha Iqbal, Sana Samreen, Wajid Syed, Hira Khan, Mahmood Basil A. Al-Rawi.

**Software:** Muhammad Mamoon Iqbal, Ayesha Iqbal, Sana Samreen, Wajid Syed, Hira Khan, Mahmood Basil A. Al-Rawi.

**Supervision:** Atif Ali, Rashida Bibi, Muhammad Mamoon Iqbal, Ayesha Iqbal, Hira Khan.

**Validation:** Marium Ayaz, Rashida Bibi, Muhammad Mamoon Iqbal, Ayesha Iqbal, Sana Samreen, Hira Khan, Mahmood Basil A. Al-Rawi.

**Visualization:** Marium Ayaz, Rashida Bibi, Muhammad Mamoon Iqbal, Ayesha Iqbal, Hira Khan.

**Writing – original draft:** Marium Ayaz, Atif Ali, Ayesha Iqbal, Wajid Syed, Hira Khan.

**Writing – review & editing:** Atif Ali, Sana Samreen, Wajid Syed, Hira Khan, Mahmood Basil A. Al-Rawi.
